# MRI segmentation of tooth tissue in age prediction of sub-adults — a new method for combining data from the 1st, 2nd, and 3rd molars

**DOI:** 10.1007/s00414-023-03149-0

**Published:** 2023-12-26

**Authors:** Mai Britt Bjørk, Øyvind Bleka, Sigrid Ingeborg Kvaal, Tomas Sakinis, Frode Alexander Tuvnes, Heidi Beate Eggesbø, Peter Mæhre Lauritzen

**Affiliations:** 1 Institute of Clinical Dentistry, Faculty of Dentistry, University of Oslo, Postboks 1109, Blindern, 00317 Oslo, Norway; 2 Department of Forensic Sciences, Oslo University Hospital, Postboks 4950 Nydalen, OUS, Rikshospitalet, 0424 Oslo, Norway; 3 Division of Radiology and Nuclear Medicine, Oslo University Hospital, Postboks 4950 Nydalen, OUS, Ullevål, 0424 Oslo, Norway; 4 Institute of Clinical Medicine, Faculty of Medicine, University of Oslo, Postboks 4950 Nydalen, OUS, 0424 Oslo, Norway; 5 Faculty of Health Sciences, Department of Life Sciences and Health, Oslo Metropolitan University, Postboks 4, St. Olavs Plass, 0130 Oslo, Norway

**Keywords:** Age determination, Sub-adults, Combining molars, MRI, Youth sports

## Abstract

**Purpose:**

We aimed to establish a model combining MRI volume measurements from the 1st, 2nd and 3rd molars for age prediction in sub-adults and compare the age prediction performance of different combinations of all three molars, internally in the study cohort.

**Material and method:**

We examined 99 volunteers using a 1.5 T MR scanner with a customized high-resolution single T2 sequence. Segmentation was performed using SliceOmatic (Tomovision©). Age prediction was based on the tooth tissue ratio (high signal soft tissue + low signal soft tissue)/total. The model included three correlation parameters to account for statistical dependence between the molars. Age prediction performance of different combinations of teeth for the three molars was assessed using interquartile range (*IQR*).

**Results:**

We included data from the 1st molars from 87 participants (F/M 59/28), 2nd molars from 93 (F/M 60/33) and 3rd molars from 67 (F/M 45/22). The age range was 14–24 years with a median age of 18 years. The model with the best age prediction performance (smallest *IQR*) was 46–47-18 (lower right 1st and 2nd and upper right 3rd molar) in males. The estimated correlation between the different molars was 0.620 (46 vs. 47), 0.430 (46 vs. 18), and 0.598 (47 vs. 18). *IQR* was the smallest in tooth combinations including a 3rd molar.

**Conclusion:**

We have established a model for combining tissue volume measurements from the 1st, 2nd and 3rd molars for age prediction in sub-adults. The prediction performance was mostly driven by the 3rd molars. All combinations involving the 3rd molar performed well.

**Supplementary Information:**

The online version contains supplementary material available at 10.1007/s00414-023-03149-0.

## Introduction

Age prediction in sub-adults may be appropriate in issues concerning migration, child marriages or criminal law. However, there is increased attention to the need for age verification in youth elite sports. To maintain fair play and to promote health and safety in youth sports, young athletes must compete with peers in age-specific events [1].

It is recommended that predicting age in living individuals is performed by evaluation of the growth and development on radiographs of the teeth, a physical examination, a radiograph of the hand and/or CT of the clavicles [2].

The skeletal maturity of young athletes may be affected by growth and endocrinological disorders, malnutrition, chronic overload and even the use of anabolic steroids. Such factors have little effect on tooth development [3, 4].

The International Olympic Committee (IOC) has recommended using MRI of third molars for predicting age in cases of suspected age fraud [1]. For children and sub-adults, MRI may be preferred to other imaging modalities, since it is radiation-free, and post-processing tools allow for extraction of high-precision data. Image data analysis increases objectivity compared to subjective grading of the different development stages.

Including data from more than one tooth or from other physical traits may increase accuracy [5].

We have previously shown that the ratio (high signal soft tissue + low signal soft tissue)/total from full volume MRI-segmentation of the 3rd molars may be a valuable parameter in age estimation [6]. In a follow-up study using the same material, we found the same ratio in the 1st and 2nd molars to be valuable and provided a novel method that managed to combine the information from the two molars [7]. Based on this, we found that the combination of the lower right teeth (46 and 47) did not increase the prediction performance much compared to using the best tooth alone (tooth 46).

In this study, we expand the methodology to enable the combination of all three different molars (1st, 2nd and 3rd) to provide age assessment [6, 7].

Our aim was first to establish a model for combining MRI tissue volume measurements from the 1st, 2nd and 3rd molars in age prediction of sub-adults. Secondly, it was to compare the age prediction performance of different combinations of all three molars, internally in the study cohort.

## Material and method

The study was approved by *the Data Protection Officer* (PVO), at Oslo University Hospital, and performed in accordance with the Declaration of Helsinki.

We included the same cohort of 99 healthy volunteers (F/M 65/34, age range 14–24 years, median 18 years) from sports clubs and universities in the period of 2018–2021, including the same imaging and segmentation data set as in two previous studies [6, 7].

All participants signed informed consent. For participants under the age of 17 years, the consent was signed by a legal trustee.

### Inclusion and exclusion criteria

Inclusion criteria were individuals from 14 to 24 years, and there were no contraindications to MRI acquisition according to the checklist from The Norwegian Directorate of Health 2017.

Exclusion criteria for the individual molars were caries, dental fillings, erosion, excessive abrasion, incorrect stabilization of the bite with dental cotton rolls and disturbing artefacts from movement or metal retainers.

### MRI acquisition

The MRI examinations were conducted on a 1.5 T scanner (Avantofit, Siemens, Erlangen, Germany) with a bilateral surface coil (Head Neck 20 and Flex Small 4 used in combination).

We used a short (5 min and 4 s) T2 3D TSE sequence yielding 0.37 mm iso-voxels [6, 7].

Two cotton rolls size 2, soaked in 2 ml of water, were placed bilaterally between the molars to displace air for better delineation of the teeth and to stabilize the bite.

### Segmentation

The MRI examinations were separated into upper (maxillary) and lower (mandibular) teeth. Semi-automated segmentation, i.e. manually using T2 signal intensity thresholds, of the 1st, 2nd and 3rd molars: 16–17-18 (upper right), 26–27-28 (upper left), 36–37-38 (lower left) and 46–47-48 (lower right), was performed on axial images using SliceOmatic (Tomovision©, Canada). The tissue volumes were calculated in ml (cm^3^).

Lower and upper thresholds were set at 0 and 63 for hard tooth tissue (dentine, enamel and cementum), 64 and 100 for low signal soft tissue and ≥ 101 for high signal soft tissue.

Two experienced forensic dentists and an experienced head and neck radiologist performed a consensual ground-truth segmentation of the first five participants. The remaining segmentations were performed by one of the experienced forensic dentists according to the method established in consensus.

The apex of the root was defined as the point at which hard tooth tissue was identifiable at least on two sides, and no segmentation was performed beyond this point.

### Statistical analyses

In this paper, we explored the age prediction performance by combining different teeth for the three different molars: 1st, 2nd and 3rd.

For this analysis, we used the natural logarithm of the response measurement (high signal soft tissue + low signal soft tissue)/total for all teeth since this measurement showed a strong linear association with chronological age. The Akaike information criterion (AIC) was used to decide the model configuration of how the sex variable, common or different age slope, and the variance weight were incorporated into the per tooth model, as shown in Table 1.

**Table 1:** Overview of the best models for 1st, 2nd, and 3rd molars separately. Performance of the best single tooth was assessed based on the *p*-value of the age variable, combined, or separate for each sex, depending on the selected model. Akaike information criterion (AIC) was used to select the model type for sex and variance weighting

Molar	Tooth	Model	Variance weighting
1st	16	Age + gender (fender as intercept)	1/age
	26	Age + gender (gender as intercept)	1/age
	36	Age + gender (gender as intercept)	1/age
	46	Age × gender (different age slopes and intercept for sex)	1/age
2nd	17	Age + gender (gender as intercept)	1
	27	Age + gender (gender as intercept)	1
	37	Age:gender (different age slopes for sex but common intercept)	1/age
	47	Age:gender (different age slopes for sex but common intercept)	1/age
3rd	18	Age × gender (different age slopes and intercept for sex)	1
	28	Age + gender (gender as intercept)	1
	38	Age (Gender is common)	Age
	48	Age (Gender is common)	Age

Additionally, we defined and built a model, which enables the combination of all three molars as demonstrated in the Appendix. This model includes three correlation parameters to account for the statistical dependence between the 1st and 2nd, the 1st and 3rd and the 2nd and 3rd molars.

In this study, we built and explored the performance of models based on different combinations of the three molars: First, the best 1st, and 2nd molars (tooth 46 and 47, found from previous study) were combined with each of the 3rd molars 18, 28, 38 and 48. Then, we explored the three molars in each quadrant: (16–17-18), (26–27-28), (36–37-38) and (46–47-48). We also compared the performance of sub-combinations of two of the three molars and of single teeth.

**Fig. 1:**
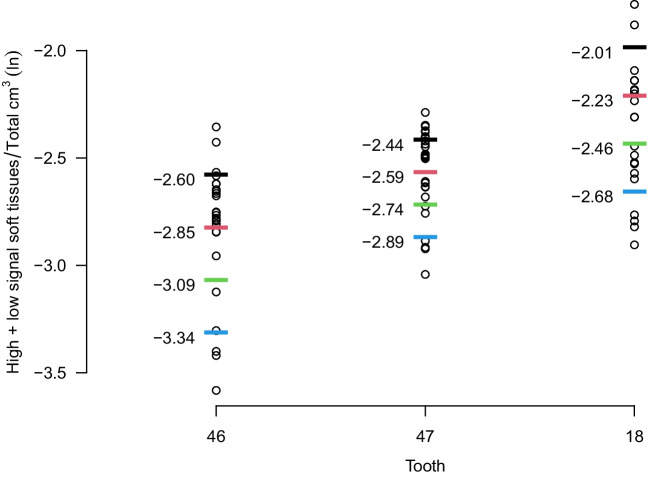
The natural logarithm (ln) of the volumes (high signal soft tissue + low signal soft tissue/total) and the four hypothetical observations for each tooth from the best model: 46, 47 and 18

We applied a Bayesian approach to provide the posterior age distributions based on the built models, where we assumed a uniform prior of age from 14 to 23 years. Four hypothetical observations for the Bayesian analysis were constructed based on the data points of each separate tooth and sex: Six points were first positioned with uniform intervals from the minimum to the maximum observation. The four interior points (not min or max) were chosen as hypothetical observations and categorised as black, red, green and blue, as shown in Fig. 1.

**Fig. 2:**
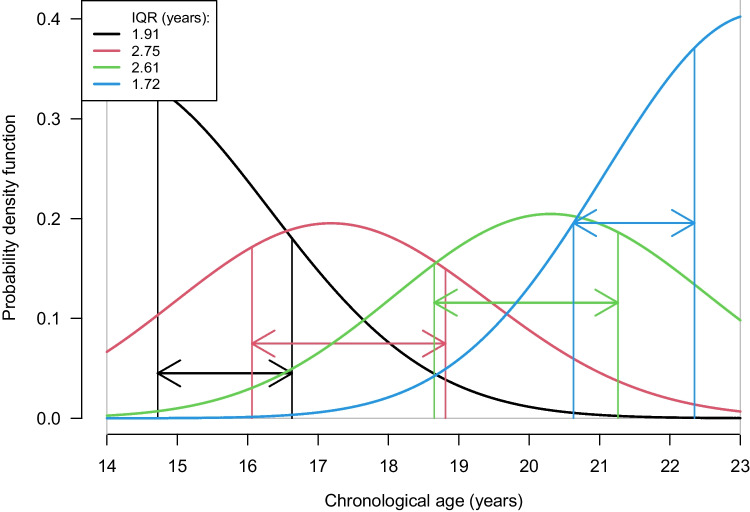
The interquartile range (*IQR*), defined as the length between the 25th and 75th percentiles, is shown with arrows for each of the color-coded curves of the posterior age distribution

We used the interquartile range (*IQR*) as a measure to assess the performance between different combinations and sub-combinations of the three molars: This measure is defined as the length between the 25th and 75th percentiles of the posterior age distribution, with chronological years as a unit, as shown in Fig. 2. A smaller *IQR* means that the age prediction performance is better. Additionally, the probability of being above 18 years old was calculated based on the posterior age distribution.

Analysis was performed using R*v*4.2.1. An R script was created to estimate the parameters using maximum likelihood estimation for building the combined models. Another script was created to perform the Bayesian age predictions for given response measurements, presented as posterior age distribution curves. *IQR* and probabilities were calculated from these curves.

## Results

### Participants

We recruited 99 healthy volunteers. After exclusion, we acquired the 1st molar data from 87 participants (F/M 59/28), 2nd molar data from 93 participants (F/M 60/33) and 3rd molar data from 67 participants (F/M 45/22). The number of observations for each molar and sex and the hypothetical observations are displayed in Table 2. Importantly, many individuals were not registered with complete sets of teeth. The number of included teeth in the different tooth combinations is summarized in Supplementary Table 1.

**Table 2:** Number of observations (*n*) for each sex and molar and the natural logarithm of the four hypothetical observations black, red, green, and blue

Sex	Tooth	*n*	Black	Red	Green	Blue
Male	16	26	− 2.57	− 2.76	− 2.96	− 3.15
	17	33	− 2.32	− 2.51	− 2.7	− 2.88
	18	21	− 2.01	− 2.23	− 2.46	− 2.68
	26	26	− 2.51	− 2.69	− 2.87	− 3.06
	27	30	− 2.29	− 2.45	− 2.6	− 2.75
	28	19	− 1.99	− 2.16	− 2.34	− 2.51
	36	26	− 2.45	− 2.66	− 2.87	− 3.08
	37	26	− 2.33	− 2.47	− 2.62	− 2.76
	38	20	− 2.03	− 2.23	− 2.42	− 2.62
	46	25	− 2.6	− 2.85	− 3.09	− 3.34
	47	29	− 2.44	− 2.59	− 2.74	− 2.89
	48	21	− 1.83	− 2.1	− 2.37	− 2.63
Female	16	56	− 2.66	− 2.83	− 3	− 3.17
	17	56	− 2.5	− 2.67	− 2.84	− 3.02
	18	41	− 2.27	− 2.41	− 2.56	− 2.71
	26	58	− 2.58	− 2.75	− 2.91	− 3.08
	27	58	− 2.48	− 2.65	− 2.81	− 2.98
	28	40	− 1.98	− 2.22	− 2.45	− 2.69
	36	53	− 2.69	− 2.88	− 3.06	− 3.24
	37	55	− 2.48	− 2.67	− 2.85	− 3.03
	38	38	− 2.04	− 2.25	− 2.47	− 2.68
	46	50	− 2.82	− 3.01	− 3.2	− 3.4
	47	56	− 2.5	− 2.66	− 2.82	− 2.98
	48	40	− 1.99	− 2.22	− 2.46	− 2.69

### Best tooth combination

Comparing models for both sexes, the best model for age prediction was the combination 46–47-18 in males, and all models reported for comparison are for males.

The *IQR* for the four hypothetical observations coloured with black (1.91 years), red (2.75 years), green (2.61 years) and blue (1.72 years) are displayed in Table 3.

**Table 3:** Inter-quartile range (*IQR*) and the probability of being older than 18 years for the four hypothetical observations black, red, green, and blue. Data from the models combining tooth 46 and 47 in males with the 3rd molar in each quadrant: 18, 28, 38, and 48

Tooth combination	Hypothetical observation	25 percentile (years)	75 percentile(years)	*IQR* (years)	Probability (%)
46–47-18	Black	14.72	16.63	1.91	8.1
	Red	16.06	18.81	2.75	39
	Green	18.65	21.26	2.61	84
	Blue	20.63	22.35	1.72	98
46–47-28	Black	14.84	17.06	2.22	13
	Red	16.08	19.06	2.98	42
	Green	18.33	21.17	2.84	80
	Blue	20.20	22.21	2.01	96
46–47-38	Black	15.03	17.58	2.55	20
	Red	16.67	19.96	3.29	55
	Green	19.00	21.72	2.72	87
	Blue	20.64	22.41	1.77	98
46–47-48	Black	14.63	16.44	1.81	6.6
	Red	15.81	18.63	2.82	35
	Green	18.30	21.19	2.89	79
	Blue	20.38	22.31	1.93	97

**Fig. 3:**
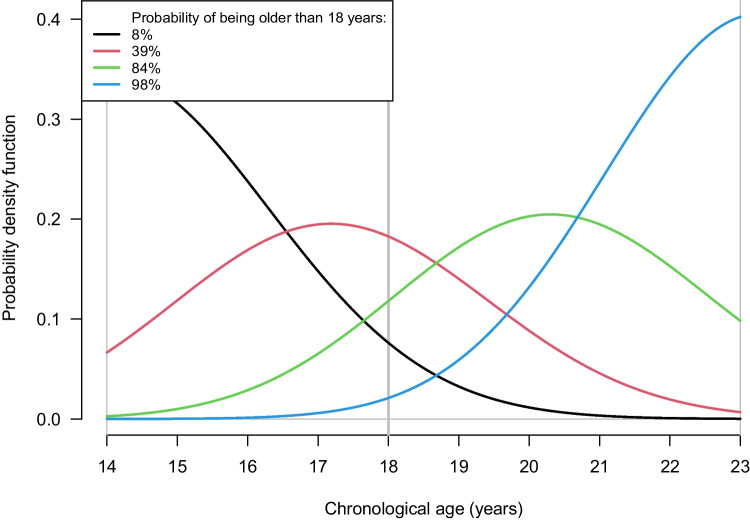
The posterior age distributions for (high signal soft tissue + low signal soft tissue)/total for the combination 46–47-18 in males. The solid curves colour-coded black, red, green and blue correspond to the natural logarithm of the four hypothetical observations. The limits of the prior age distribution (14.0 and 23.0 years) and the 18-year threshold are shown as vertical, solid grey lines

For this tooth combination, 46–47-18, the probability of being older than 18 years for the four different colour-coded curves in Fig. 3 is 98% (blue), 84% (green), 39% (red) and 8% (black). All the different tooth combinations in each sex, *IQR* results and probabilities of being above 18 years are shown in Supplementary Table 2.

The age prediction performance of each of the individual teeth and different combinations of these three teeth 46, 47 and 18, in males are shown in Table 4. For the hypothetical observation with the highest probability of being older than 18 years (colour-coded blue) the probability ranged from 94 to 99% and *IQR* from 1.67 to 2.22 years for the different combinations of teeth.

**Table 4:** Inter quartile range (*IQR*) and the probability of being older than 18 years in each combination of the 1st, 2nd, and 3rd molars from the best model: 46, 47, and 18 in males and the four hypothetical observations with the colors black, red, green, and blue

Tooth combination	Hypothetical observation	*IQR* (years)	Probability (%)
1st	Black	3.35	32
2nd		3.65	39
3rd		2.09	11
1st and 2nd		3.18	29
1st and 3rd		2.02	9.8
2nd and 3rd		2.03	10
1st, 2nd, and 3rd		1.91	8.1
1st	Red	3.82	57
2nd		3.81	61
3rd		2.87	42
1st and 2nd		3.70	58
1st and 3rd		2.81	43
2nd and 3rd		2.81	40
1st, 2nd, and 3rd		2.75	39
1st	Green	3.08	82
2nd		3.07	82
3rd		2.71	83
1st and 2nd		2.91	85
1st and 3rd		2.58	86
2nd and 3rd		2.68	83
1st, 2nd, and 3rd		2.61	84
1st	Blue	2.09	95
2nd		2.22	94
3rd		1.84	97
1st and 2nd		1.94	97
1st and 3rd		1.67	99
2nd and 3rd		1.84	98
1st, 2nd, and 3rd		1.72	98

The best tooth combination excluding 3rd molars was 46–47. For the hypothetical observation with the highest probability of being older than 18 years (colour-coded blue) the probability was 97% with an *IQR* of 1.94 years.

The model including all three molars, with the lowest performance in males, was 26–27-28. For the hypothetical observation with the highest probability of being older than 18 years (colour-coded blue) the probability was 85%, with an *IQR* of 2.73 years.

### Comparison of the contribution of each molar

**Figs. 4:**
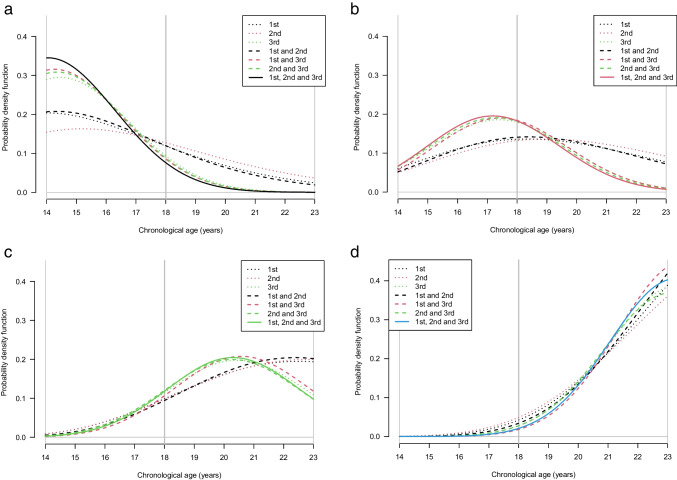
**a**, **b**, **c**, and **d** Age distribution curves for the four hypothetical observations in different combinations of the molars: 46, 47 and 18 in males: black line (**a**), red line (**b**), green line (**c**) and blue line (**d**). The different combinations of the 1st, 2nd and 3rd molars are labelled with dashed, dotted or solid lines in different colours

The posterior age distributions and hence the prediction performance was mostly driven by the 3rd molars for all tooth combinations, as shown in Fig. 4 a, b, c and d.

**Fig. 5:**
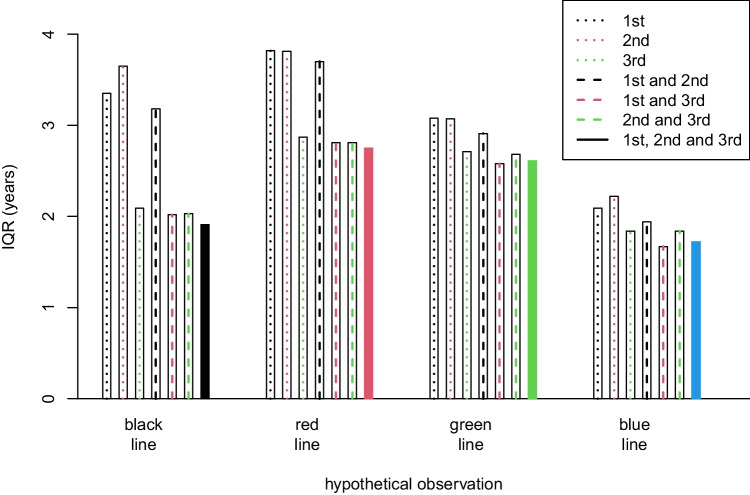
Age prediction performance of different combinations of the three molars from the best model: 46, 47 and 18, in males, measured as the interquartile range (*IQR*) and grouped according to the four hypothetical observations (black, red, green and blue). Each column represents a different combination of the 1st, 2nd and 3rd. The *IQR* values and the probability of being older than 18 years for the four hypothetical observations are shown in Table 4

The *IQR* of all combinations of teeth from the best model 46, 47 and 18 in males are displayed in Fig. 5, corresponding to the colour-coded curves in Fig. 4 a, b, c and d. For all hypothetical observations, the *IQR* was the smallest in tooth combinations including a 3rd molar. An overview of the *IQR* for different combinations in each sex is shown in Supplementary Fig. 1 a and b. The *IQR* for other tooth combinations was in general higher, though the differences are sometimes not very large.

### Estimated correlations between the molars

Parameter estimates for the best model, 46–47-18 are shown in Supplementary Table 3. The maximum likelihood estimate of the correlation parameters was 0.620 (46 vs. 47), 0.430 (46 vs. 18) and 0.598 (47 vs. 18).

## Discussion

To our knowledge, this is the first in vivo study to calculate the total tooth tissue volumes and to establish an age prediction model combining data from all three molars, taking dependency between molars into account. Age prediction performance was mostly driven by the 3rd molars, and all models including a 3rd molar performed well.

### Best tooth combination

We found that the lower right 1st and 2nd molars and upper 3rd molar (46–47-18) in males provided the best predictive model since in general, this tooth combination obtained a smaller *IQR* than for other combinations.

This result matches the results in our previous research where tooth 46, 47 and 18 were the best performing single teeth in males [6, 7]. The prediction performance was mostly driven by the 3rd molars, and all combinations involving the 3rd molar consistently demonstrated smaller *IQR* values, regardless of the quadrant of the 3rd molar. The explanation may be that the 3rd molar is the only tooth still developing after the age of 18 years. This highlights the importance of incorporating data from the 3rd molars when utilizing MRI segmentation of tooth tissue for age predictions in sub-adults.

The strong performance across various tooth combinations shows the model’s robustness to missing teeth and adaptability in the likely event that another tooth combination shows better performance in another cohort.

### Comparison of the contribution of each molar

The performance of the other models, shown in Supplementary Table 2 and Supplementary Fig. 1 a and b, shows that other tooth combinations may be used if the 3rd molars are missing.

Several models combining only the 1st and 2nd molars also performed well. For the best model, 46–47-18, the probability of being older than 18 years increased by only 1% (from 97 to 98%), and the *IQR* decreased from 1.94 to 1.72 years by adding the 3rd to the 1st and 2nd molars. However, other 1st and 2nd molar combinations had lower performance than 46–47. Nevertheless, our method has the advantage of being robust to agenesis of one or more 3rd molars, the rate of which ranges from 5 to 56% in different studies [8].

### Statistical dependence between molars

Our age prediction model includes three correlation parameters to account for the statistical dependence between the three molars. We found a considerable dependency between the different molars, with the highest being between the 1st and 2nd molars, and the smallest between the 1st and 3rd molars. This is in accordance with the fact that the 1st and 2nd molars are closest in development, while the 1st and 3rd molars are further apart.

### 3D imaging

Using 3D imaging, which presented the entire tooth, we found that the upper molars performed well. This is interesting since the upper molars have been studied less with conventional 2D images like orthopantomogram (OPG), since they are frequently angulated and/or superimposed on adjacent structures [9]. Segmentation tools make it possible to perform tissue volumes. Quantitative results from counting voxels are more objective and ethically defensible because of the high potential for errors and bias associated with the grading of tooth development.

Teeth are more appropriate to use because they are little affected by external factors compared with skeletal maturity [4]. Some of the volunteers were recruited from sports clubs. Elite athletes may advance or delay their skeletal development, but such overload does not affect teeth. Therefore, there was no selection bias for these participants regarding chronic overload compared to other research using skeletal traits [10].

Combining our method with other physical traits such as skeletal development and DNA methylation is an approach recommended in the legal context [5, 11, 12]. A combination might reduce the uncertainty stemming from biological variation and enhance the method’s resilience against gaps in teeth data caused by missing teeth. Nevertheless, the most effective combination of dental and other physical traits for accurate age prediction requires further exploration.

### Bayesian approach

Utilizing Bayesian modelling is advisable, as it offers greater resilience against age distribution influences originating from both the reference and target samples [13]. The Bayesian framework is (after all) an illustration of how one can proceed with data to describe the uncertainty and not just expectation (an extension of ordinary regression) [7].

The upper and lower prior were identical to our two prior studies [6, 7]. The lower age prior was dictated by the 3rd molar development, as the 3rd molar is less suitable for age prediction in individuals below 14 years of age. However, the upper prior was a matter of choice and was set at 23 years. Increasing the upper prior increases the risk of falsely classifying an individual as older than 18 years.

### Limitations

Our study group was relatively small with limited ethnic diversity, and our method needs validation in an independent cohort before it can be applied [14]. Therefore, we have yet to determine the potential of our approach in narrowing age prediction intervals, alone, or in combination with other physical traits.

The volunteers did not exhibit excessive tooth wear, caries or dental fillings. As a result, our methodology might not yield optimal results among individuals with a lower socioeconomic status [15–17]. Ideally, all participants would have possessed their full set of the twelve molars for comprehensive presentation. Nevertheless, due to exclusion criteria and instances of 3rd molar agenesis, this complete representation was unattainable, as in a “real world” situation.

## Conclusion

We have established a model for combining tissue volume measurements from the 1st, 2nd and 3rd molars for age prediction in sub-adults. The prediction performance was mostly driven by the 3rd molars. All combinations involving the 3rd molar performed well.

### Electronic supplementary material

Below is the link to the electronic supplementary material.

Supplementary file1 (PDF 10 KB) Interquartile range (IQR) for the different combinations where tooth 46 and 47 are combined with the four third molars (18, 28, 38, and 48), and for the three molars within each quadrant. The different combinations of the 1st, 2nd, and 3rd molars in males **a)** and females **b)**, and the four hypothetical observations: black, red, green, and blue. Each molar combination has its color as shown in the lower right corner.

Supplementary file2 (PDF 10 KB)

Supplementary file3 (PDF 17 KB)

Supplementary file4 (PDF 366 KB)

Supplementary file5 (PDF 10 KB)

## Data Availability

All the data were registered, including the data that was deleted or changed. Anonymized data were exported for statistical calculations. After the database lock, the data was saved according to current regulations.

## Appendix: Mathematical description for the combination model

### The likelihood function: Used for estimating model parameters

We implemented a model to combine the information of three methods, in such a way that all individuals are utilized in the estimation of the parameters, even if they have missing values for one of the methods. This can be done by splitting the likelihood function into separate parts:

$$L\left(\theta \right)= {[L}_{1}\left({\theta }_{1}\right){L}_{2}\left({\theta }_{2}\right){L}_{12}\left({\theta }_{12}\right){L}_{1}\left({\theta }_{1}\right)]{[L}_{3}\left({\theta }_{3}\right){L}_{13}\left({\theta }_{13}\right){L}_{23}\left({\theta }_{23}\right){L}_{123}\left({\theta }_{123}\right)]$$

${L}_{1}\left({\theta }_{1}\right){L}_{2}\left({\theta }_{2}\right){L}_{3}\left({\theta }_{3}\right){L}_{ij}\left({\theta }_{ij}\right)ij{L}_{123}\left({\theta }_{123}\right)$, and are the marginal likelihood expressions for the three methods, where the individuals are only recorded with measurements for either of these methods. is the likelihood based on the bivariate normal model where individuals are recorded only with the combination of method and. Last, is the likelihood of a trivariate normal model where individuals are recorded for all three methods.

The unknown parameters consist of $\theta =({\beta }_{1},{\sigma }_{1},{\beta }_{2},{\sigma }_{2},{\beta }_{3},{\sigma }_{3},{\rho }_{12},{\rho }_{13},{\rho }_{23})$: $\beta$ includes both the intercept and slope parameters (and possibly additional for each sex), $\sigma$ is the standard deviation, with index indicating the method and $\rho$ is the correlation parameter between two methods (pairwise). The subscripts indicate which method or pair of methods the parameters belong. We estimated the unknown parameters $\theta$ using maximum likelihood estimation:  $\widehat{\theta }=argma{x}_{\theta }L\left(\theta \right).$ Further follows more details about the underlying model.

### The underlying model for observations: Used to define the likelihood function

We define ${Y}_{m}$ as the stochastic variable for the response variable (transformed tooth volume measurement) of method $m$, with individual observations for individual $i$ given as ${y}_{i,m}$. By inserting the response variable for individual of method $m$ into the probabilistic model (defined below) we obtain the likelihood function as

$${L}_{m}\left({\theta }_{m}\right)=\prod_{i=1}^{{n}_{m}}P\left({Y}_{m}={y}_{i,m }\right|{\theta }_{m})$$

where ${n}_{m}$ is the number of individuals recorded only for a method $m$. The likelihood function for the bivariate joint model (method $k$ and $m$) is given as

$${L}_{km}\left({\theta }_{km}\right)=\prod_{i=1}^{{n}_{km}}P\left({Y}_{k}={y}_{i,k },{Y}_{m}={y}_{i,m }\right|{\theta }_{km})$$

where ${n}_{km}$ is the number of individuals recorded for both methods. The likelihood function for the trivariate joint model (between all three methods) is given as.

$${L}_{123}\left({\theta }_{123}\right)=\prod_{i=1}^{{n}_{123}}P\left({Y}_{1}={y}_{i,1 },{Y}_{2}={y}_{i,2 },{Y}_{3}={y}_{i,3 }\right|{\theta }_{123})$$

where ${n}_{123}$ is the number of individuals recorded for all methods.

We further introduce the design matrix $X$ which includes additional information about the individuals: a factor for sex and a numerical value for age. We let ${X}_{i}$ be the design matrix and ${w}_{im}$ to be the specified variance weight for individual $i$ considered for method $m$.

We extend the defined probabilistic model from previous paper [8] such that the combination of all three methods follow a trivariate normal distribution (Multivariate Normal with dimension 3):

$$P\left({Y}_{1}={y}_{i,\,1 },{Y}_{2}={y}_{i,2 },{Y}_{3}={y}_{i,3 }\right|\theta )= MV{N}_{3} \left(\left[\begin{array}{c}\begin{array}{c}{y}_{i,1}\\ {y}_{i,2}\end{array}\\ {y}_{i,3}\end{array} \right], \left[\begin{array}{c}\begin{array}{c}{X}_{i}{\beta }_{1}\\ {X}_{i}{\beta }_{2}\end{array}\\ {X}_{i}{\beta }_{3}\end{array}\right],\left[\begin{array}{ccc}{\sigma }_{1}^{2}{w}_{i1}& {c}_{12}& {c}_{13}\\ {c}_{12}& {\sigma }_{2}^{2}{w}_{i2}& {c}_{23}\\ {c}_{13}& {c}_{23}& {\sigma }_{3}^{2}{w}_{i3}\end{array}\right]\right)$$

where ${c}_{km}= {{\rho }_{km}\sigma }_{k}{\sigma }_{m}{(\surd w}_{ik}){(\surd w}_{im})$ is the covariance between variable $k$ and $m$.

The marginal and bivariate models are easily extracted as subsets from this definition:

$$P\left({Y}_{m}={y}_{i,m }\right|{\theta }_{m})=N\left({y}_{i,m }|{X}_{i}{\beta }_{m},{\sigma }_{m}^{2}{w}_{im}\right)$$

$$P\left({Y}_{k}={y}_{i,k },{Y}_{m}={y}_{i,m }\right|{\theta }_{km})= MV{N}_{2} \left(\left[\begin{array}{c}{y}_{i,k}\\ {y}_{i,m}\end{array} \right], \left[\begin{array}{c}{X}_{i}{\beta }_{k}\\ {X}_{i}{\beta }_{m}\end{array}\right],\left[\begin{array}{cc}{\sigma }_{k}^{2}{w}_{ik}& {c}_{km}\\ {c}_{km}& {\sigma }_{m}^{2}{w}_{im}\end{array}\right]\right)$$

In our implementation we assume that the variance weights are selected based on AIC model selection.:

### Prediction of new individuals utilizing trivariate measurements

We used the estimated maximum likelihood parameters as fixed parameters to predict age of a new individual. From previous section we defined a joint model for observed measurements on three methods (${y}_{1}$, ${y}_{2}$ and ${y}_{3}$): Since age is an independent variable as part of the design matrix $X$ we have that, for a given sex,

$$P\left({Y}_{1}={y}_{1 },{Y}_{2}={y}_{2 },{Y}_{3}={y}_{3 }\right|\theta )=P\left({Y}_{1}={y}_{1 },{Y}_{2}={y}_{2 },{Y}_{3}={y}_{3 }\right|\theta ,Age=a)$$

By applying Bayes theorem, we can obtain an expression for the posterior distribution of age:

$$P\left(Age=a\right|{y}_{1 },{y}_{2},{y}_{3})= c*P\left({Y}_{1}={y}_{1 },{Y}_{2}={y}_{2 },{Y}_{3}={y}_{3 }\right|\theta =\widehat{\theta },Age=a)P(Age=a)$$

where $P(Age=a)$ is the prior distribution of age for a new predicting individual, and $c$ is a normalization constant.

## Abbreviations

AIC: Akaike information criterion
IOC: International Olympic Committee
*IQR*: Interquartile range
CT: Computed tomography
MRI: Magnetic resonance imaging
OPG: Orthopantomogram
TSE: Turbo spin echo
T2: Transverse relaxation time
16: Upper right 1st molar
26: Upper left 1st molar
36: Lower left 1st molar
46: Lower right 1st molar
17: Upper right 2nd molar
27: Upper left 2nd molar
37: Lower left 2nd molar
47: Lower right 2nd molar
18: Upper right 3rd molar
28: Upper left 3rd molar
38: Lower left 3rd molar
48: Lower right 3rd molar
