# Supplementary figures and images for: MRI segmentation of tooth tissue in age prediction of sub-adults — a new method for combining data from the 1st, 2nd, and 3rd molars
Source: Int J Legal Med. 2023 Dec 26;138(3):939–49. doi: 10.1007/s00414-023-03149-0 (PMC11003927; doi:10.1007/s00414-023-03149-0)

IQR

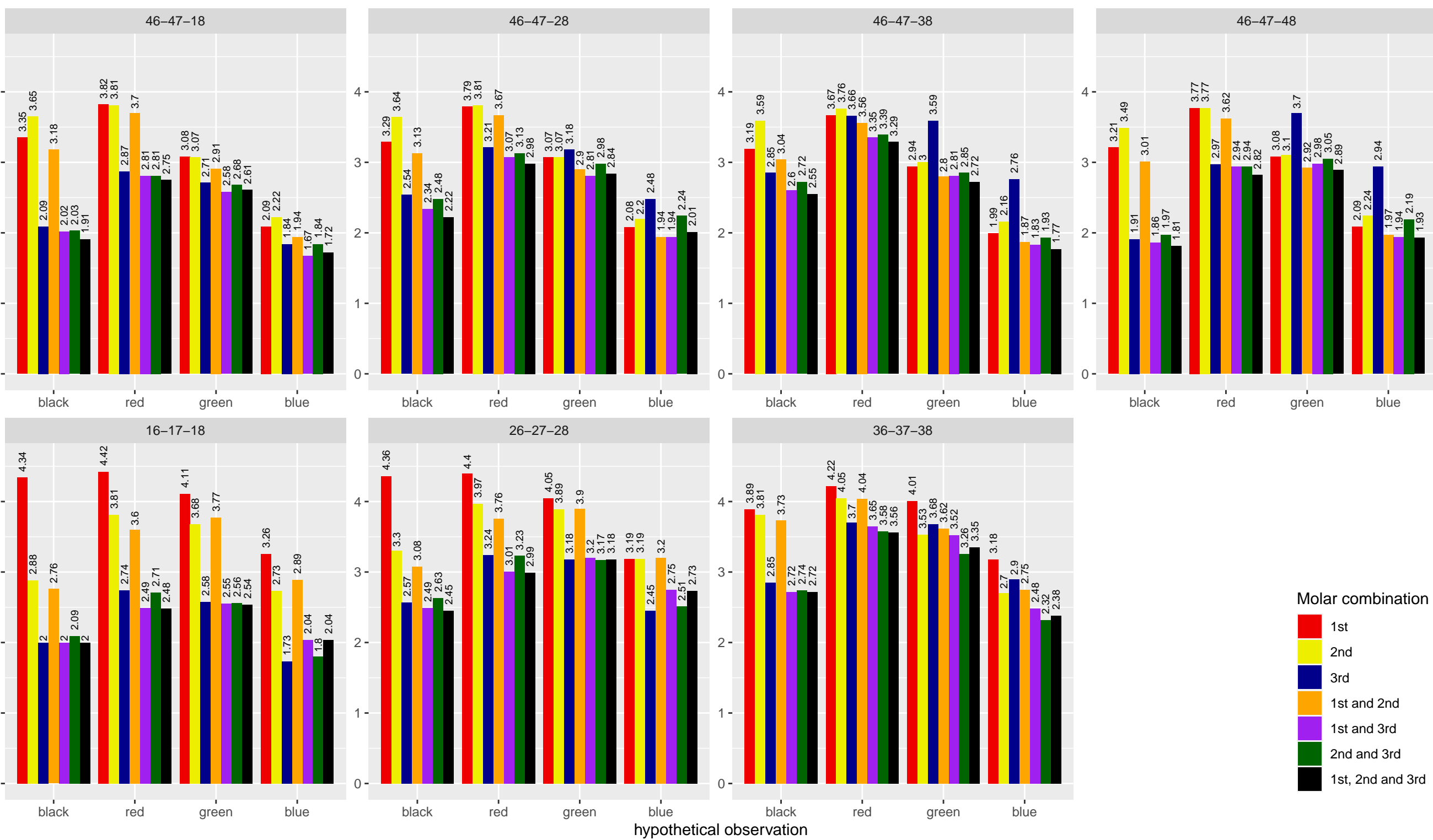

Supplement: Supplementary file 1 — Supplementary file1 (PDF 10 KB) Interquartile range (IQR) for the different combinations where tooth 46 and 47 are combined with the four third molars (18, 28, 38, and 48), and for the three molars within each quadrant. The different combinations of the 1st, 2nd, and 3rd molars in males a) and females b), and the four hypothetical observations: black, red, green, and blue. Each molar combination has its color as shown in the lower right corner. [file 414_2023_3149_MOESM1_ESM.pdf]

IQR

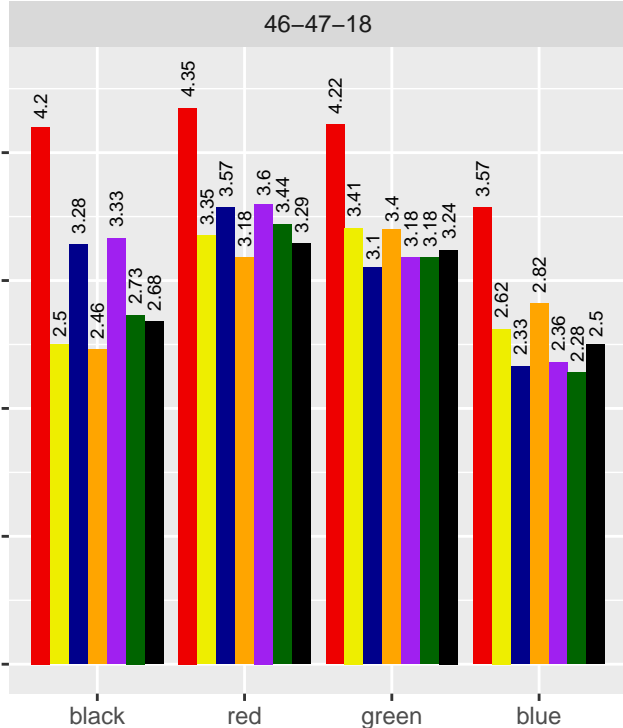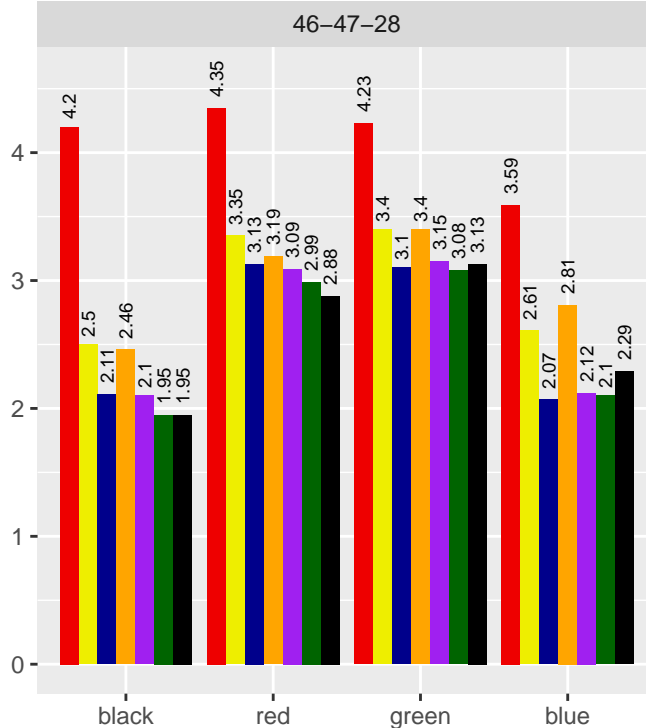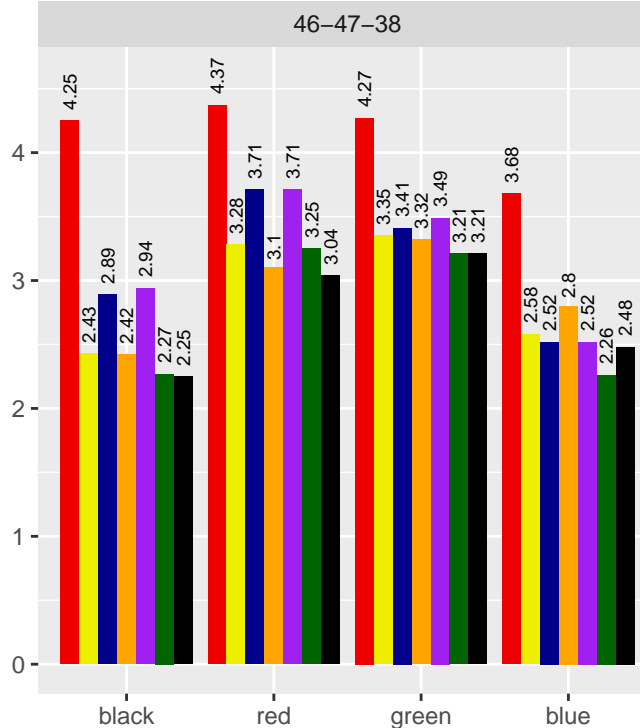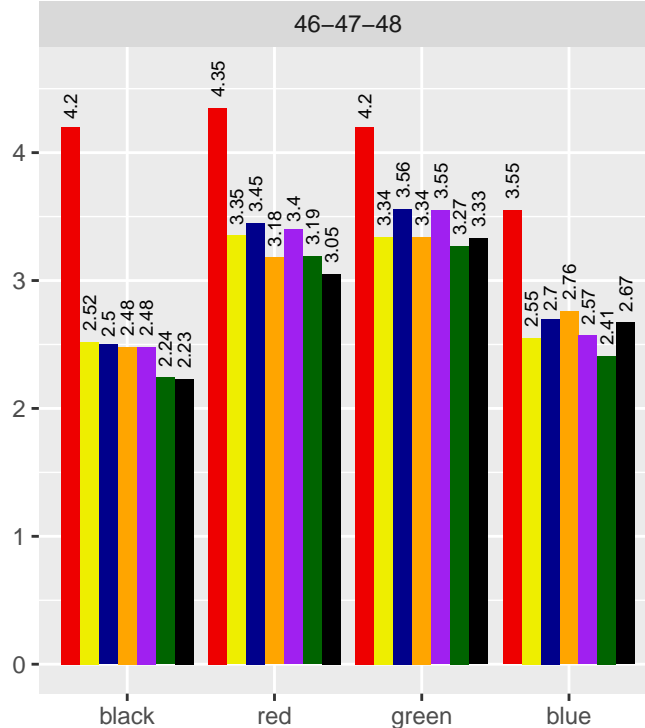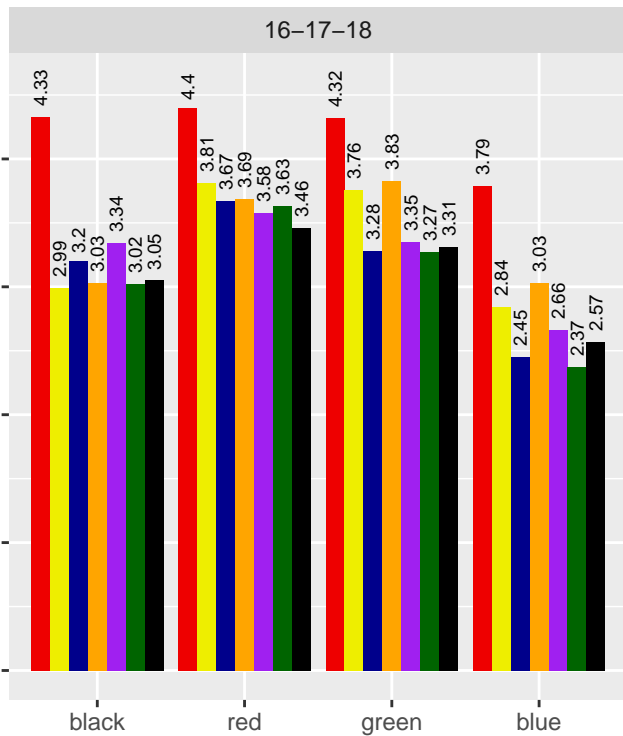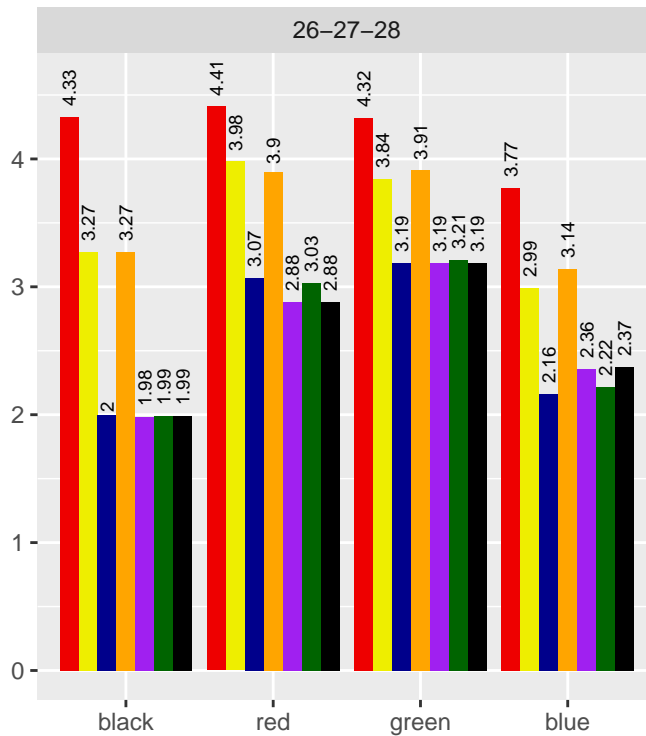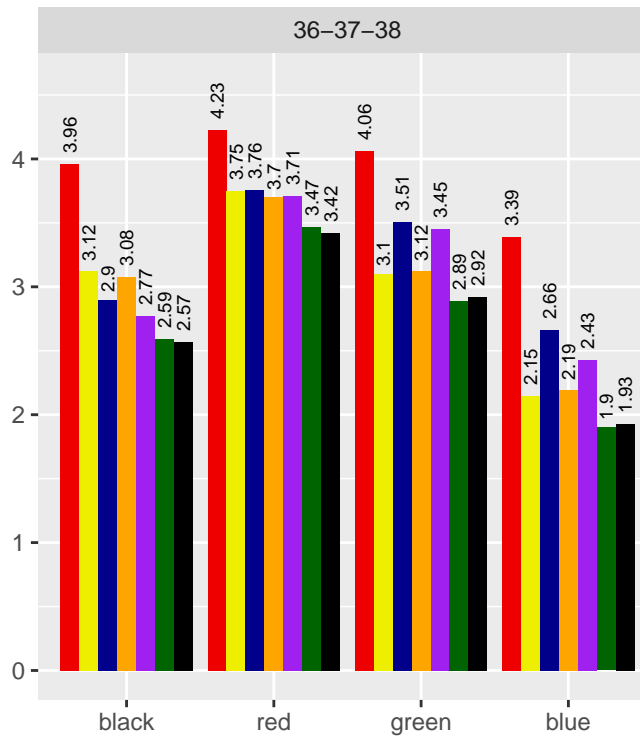

Molar combination

- 1st
- 2nd
- 3rd
- 1st and 2nd
- 1st and 3rd
- 2nd and 3rd
- 1st, 2nd and 3rd

hypothetical observation

Supplement: Supplementary file 2 — Supplementary file2 (PDF 10 KB) [file 414_2023_3149_MOESM2_ESM.pdf]
