# Supplementary material for: MRI segmentation of tooth tissue in age prediction of sub-adults — a new method for combining data from the 1st, 2nd, and 3rd molars
Source: Int J Legal Med. 2023 Dec 26;138(3):939–49. doi: 10.1007/s00414-023-03149-0 (PMC11003927; doi:10.1007/s00414-023-03149-0)
Supplement: Supplementary file 3 — Supplementary file3 (PDF 17 KB) [file 414_2023_3149_MOESM3_ESM.pdf]

Supplementary Table 1 Total number of contributing individuals in each sex and counts of each of the three molars for the combinations where tooth 46 and 47 are combined with a 3rd molar in each quadrant: 18, 28, 38, and 48 and the combinations of the 1st, 2nd, and 3rd molar within each quadrant.

		One molar only			Two molars, combined			All three molars
**Combination**	**Total**	**1st**	**2nd**	**3rd**	**1st and 2nd**	**1st and 3rd**	**2nd and 3rd**	**1st, 2nd, and 3rd**
**Male**								
46-47-18	33	2	4	1	6	1	3	16
46-47-28	33	3	3	1	8	0	4	14
46-47-38	33	2	3	1	8	1	4	14
46-47-48	33	2	3	1	7	1	4	15
16-17-18	34	0	3	1	10	0	4	16
26-27-28	33	1	2	2	11	0	3	14
36-37-38	33	3	3	2	7	2	2	14
46-47-48	33	2	3	1	7	1	4	15
**Female**								
46-47-18	59	1	3	1	14	1	5	34
46-47-28	59	1	3	1	15	1	5	33
46-47-38	58	1	4	0	15	1	4	33
46-47-48	59	1	2	1	16	1	6	32
16-17-18	61	2	3	2	15	1	0	38
26-27-28	61	1	1	2	19	0	0	38
36-37-38	59	1	3	0	17	3	3	32
46-47-48	59	1	2	1	16	1	6	32
