# Supplementary material for: MRI segmentation of tooth tissue in age prediction of sub-adults — a new method for combining data from the 1st, 2nd, and 3rd molars
Source: Int J Legal Med. 2023 Dec 26;138(3):939–49. doi: 10.1007/s00414-023-03149-0 (PMC11003927; doi:10.1007/s00414-023-03149-0)
Supplement: Supplementary file 5 — Supplementary file5 (PDF 10 KB) [file 414_2023_3149_MOESM5_ESM.pdf]

Supplementary Table 3 The estimated parameters for the combined model for teeth 46-47-18 vs. the marginal models for the best transformation of each of the three molars. The correlation for the marginal models was calculated from the residuals.

Model	Combined	Marginal
**1st molar**		
Intercept	-1.771	-1.513
GenderF	-0.808	-1.046
GenderM : Age	-0.057	-0.072
GenderF : Age	-0.026	-0.027
Sigma	0.053	0.053
**2nd molar**		
Intercept	-1.826	-1.816
GenderM : Age	-0.039	-0.04
GenderF : Age	-0.051	-0.051
Sigma	0.038	0.038
**3rd molar**		
Intercept	-0.925	-0.738
GenderF	-0.476	-0.913
GenderM : Age	-0.075	-0.087
GenderF : Age	-0.054	-0.042
Sigma	0.176	0.174
**Correlation**		
1st vs. 2nd molar	0.620	0.577
1st vs. 3rd molar	0.430	0.377
2nd vs. 3rd molar	0.598	0.484
